# Features of *Ppd-B1* expression regulation and their impact on the flowering time of wheat near-isogenic lines

**DOI:** 10.1186/s12870-017-1126-z

**Published:** 2017-11-14

**Authors:** Antonina A. Kiseleva, Elena K. Potokina, Elena A. Salina

**Affiliations:** 1 The Federal Research Center “Institute of Cytology and Genetics of Siberian Branch of the Russian Academy of Sciences”, Prospekt Lavrentyeva 10, Novosibirsk, Russian Federation 630090; 2 N.I. Vavilov Research Institute of Plant Genetic Resources, B.Morskaya Street 42-44, St. Petersburg, Russian Federation 190000

**Keywords:** Photoperiod sensitivity, Flowering time, *Ppd-B1*, Phytochrome, Common wheat

## Abstract

**Background:**

Photoperiod insensitive *Ppd-1a* alleles determine early flowering of wheat. Increased expression of homoeologous *Ppd-D1a* and *Ppd-A1a* result from deletions in the promoter region, and elevated expression of *Ppd-B1a* is determined by an increased copy number.

**Results:**

In this study, using bread wheat cultivars Sonora and PSL2, which contrast in flowering time, and near-isogenic lines resulting from their cross, “Ppd-m” and “Ppd-w” with *Ppd-B1a* introgressed from Sonora, we investigated the putative factors that influence *Ppd-B1a* expression. By analyzing the *Ppd-B1a* three distinct copies, we identified an indel and the two SNPs, which distinguished the investigated allele from other alleles with a copy number variation.

We studied the expression of the *Ppd-A1*, *Ppd-B1a*, and *Ppd-D1* genes along with genes that are involved in light perception (*PhyA*, *PhyB*, *PhyC*) and the flowering initiation (*Vrn-1*, *TaFT1*) and discussed their interactions. Expression of *Ppd-B1a* in the “Ppd-m” line, which flowered four days earlier than “Ppd-w”, was significantly higher. We found *PhyC* to be up-regulated in lines with *Ppd-B1a* alleles. Expression of *PhyC* was higher in “Ppd-m”. Microsatellite genotyping demonstrated that in the line “Ppd-m”, there is an introgression in the pericentromeric region of chromosome 5B from the early flowering parental Sonora, while the “Ppd-w” does not have this introgression. *FHY3/FAR1* is known to be located in this region. Expression of the transcription factor *FHY3/FAR1* was higher in the “Ppd-m” line than in “Ppd-w”, suggesting that *FHY3/FAR1* is important for the wheat flowering time and may cause earlier flowering of “Ppd-m” as compared to “Ppd-w”.

**Conclusions:**

We propose that there is a positive bidirectional regulation of *Ppd-B1a* and *PhyC* with an *FHY3/FAR1* contribution.

The bidirectional regulation can be proposed for *Ppd-A1a* and *Ppd-D1a*. Using in silico analysis, we demonstrated that the specificity of the *Ppd-B1* regulation compared to that of homoeologous genes involves not only a copy number variation but also distinct regulatory elements.

**Electronic supplementary material:**

The online version of this article (10.1186/s12870-017-1126-z) contains supplementary material, which is available to authorized users.

## Background

Photoperiod sensitivity is an important agronomic trait that influences the wheat heading date, and the *Ppd-1* (*Photoperiod-1*) genes are significant regulators of this process. The *Ppd-1* genes are members of the *Pseudo-Response Regulator* (*PRR*) gene family [1]. There are three *Ppd-1* genes in the hexaploid wheat *Triticum aestivum*, which are located on the short arms of chromosomes 2A, 2B and 2D [2–4] and are designated *Ppd-A1*, *Ppd-B1* and *Ppd-D1*, respectively. Mutant alleles that are responsible for photoperiod insensitivity and, thus, early flowering in short day (SD) conditions are labeled with the suffix “a”.

To date, the photoperiod insensitive (PI) alleles of all the *Ppd-1* genes have been identified. *Ppd-D1a* has a 2089 bp deletion in its promoter region [1]. This allele is widely used in wheat selection, and its influence on the phenotype has been well studied [5, 6]. *Ppd-A1a* dominant alleles were first described using *T. durum* (*Triticum durum*). There are two alleles, both of which are characterized by 1027 bp or 1117 bp deletions in the promoters [4]. The *T. aestivum Ppd-A1a* allele, which was described by Nishida et al. [7], has a 1085 bp deletion in the 5′ UTR region similarly to the other PI alleles.

Nishida et al. [7] revealed the PI *Ppd-B1a* allele in the cultivar Winter-Abukumawase with a 308 bp insertion in the promoter region. This insertion was suggested to be a MITE (miniature inverted-repeat transposable element). Another PI *Ppd-B1* mutation is a copy number variation (CNV) [8]. The haploid copy number of the wild-type *Ppd-B1* allele is one, while the PI alleles possess two to four copies. In addition to the copy number, these alleles may have different intercopy regions. Thus, there are three variants of the *Ppd-B1a* alleles based on the differences in the copy number and the intercopy junctions as follows: Recital type, Sonora64/Timstein/C591 type and Chinese Spring type [8]. Kiss et al. [9] ascertained that the phenotypical effect of the *Ppd-B1* loci is associated not only with the copy number but also with the intercopy junction type. Under field conditions, the *Ppd-B1* intercopy junction type was demonstrated to influence the heading date more than the number of copies.

The photoperiod sensitive (PS) *Ppd-1* alleles are expressed during the light period, and the peak of the expression occurs 3 to 6 h after dawn; these alleles are not expressed during the dark period. In contrast, all PI alleles demonstrate a misexpression throughout the 24 period [1, 4, 8]. The *Ppd-1a* alleles are expressed both during the light and dark periods. During the night period, PI *Ppd-1a* alleles were shown to up-regulate *TaFT1* (*Triticum aestivum Flowering Locus T*) expression under SD (Short Day) conditions [10, 11]. *TaFT1* is expressed in the leaves and transfers to the floral meristems to induce flowering [12].

The aim of the current investigation is to study the *Ppd-B1* PI allele with an increased number of copies and characterize the functional specifications of this allele and its interaction with other photoperiod genes. PCR analysis, molecular cloning and sequencing were used for the characterization of the *Ppd-B1* sequence. To analyze the diurnal expression of genes that regulate the heading date, qPCR with SYBR Green I was performed. The PlantPAN 2.0 database [13] was used to determine the putative plant transcription factor binding sites.

## Methods

### Plant materials

Two pairs of the near-isogenic lines “Ppd-m” and its sister line “Ppd-0^m^”, “Ppd-w” and its sister line “Ppd-0^w^” and their parental forms Sonora (К-47942, *Vrn-A1/Vrn-B1/Vrn-D1* and *Ppd-A1b/Ppd-B1a/Ppd-D1b*) and PSL2 (Photoperiod Sensitive Line 2, *Vrn-A1/Vrn-B1/vrn-D1* and *Ppd-A1b/Ppd-B1b/Ppd-D1b*), which have different heading dates, were used in this investigation. Lines “Ppd-m” and “Ppd-w” differ from their sister lines by the introgressions on the 2B chromosome from the Sonora variety. This difference was shown previously using SSR analysis [14]. An analysis of specific molecular markers also demonstrated that the near-isogenic lines (NILs) have recessive alleles of *Ppd-D1* and *Ppd-A1* [14]. DNA was extracted from the seedlings using the GeneJET Plant Genomic DNA Purification Mini Kit (Thermo Fisher Scientific, Lithuania) according to the manufacturer’s protocol.

### *Ppd-B1* intercopy region analysis

Previously reported PCR primers [8] were used to define the *Ppd-B1* intercopy region. The PCR was performed as previously described [8].

### SSR genotyping

SSR genotyping of the full genome of NILs was performed in previous study [14]. Unfortunately, there was a mistake in illustration described “Ppd-w” and “Ppd-0^w^” lines. “Ppd-w” and “Ppd-0^w^” lines do not have an introgression on 5B chromosome. Therefore, to ascertain the 5B chromosome inheritance in the NILs, we used an additional set of markers (*Xgpw358*, *Xbarc74*, *Xgwm213*, *Xgpw2124*). The marker sequences and their annealing temperatures are available [15]. The amplified fragments were analyzed in 5% high-resolution agarose MS-8 (Dia-m, Russia) using ethidium bromide staining.

### Primer design, PCR and sequencing of *Ppd-B1*

Previously, we have hypothesized that the *Ppd-B1* allele in the investigated lines might be characterized by sequence mutations in addition to copy number variations [14]. To investigate all possible sequence variations, we developed primers with a coding region that is specific to all *Ppd-1* genes. The primer sequences are as follows: F2-Ppd-exon2-ACCAGGCGTGGGCGTATCT; R2-Ppd-exon6-GCTCTAGCTGCCTGTTGGG; F3-Ppd-exon6-TGGAGATAGGTGCCCCTGG; and R3-Ppd-3UTR-GGACCGTCTCTGAATGATCCA. The primers for the promoter region were *Ppd-B1* gene-specific due to the strong differences in the *Ppd-1* promoter region and were designed according to the alignment of 184 sequences from the NCBI database. The primer sequences are as follows: F-Ppd-5UTR-CACTCTTATTCCCTCTATGCC and R-Ppd-5UTR-CTGTTATTATTGGAATCGTCAG. The reaction mixture was as follows: DNA in a concentration of 5 ng/μL, 1× buffer for Taq-polymerase (pH 8.6, 2.5 mM Mg2^+^), 200 μmoles dNTPs, 0.2 μmoles of each primer, 1 U Taq polymerase (Medigen, Russia), and sterile water up to a volume of 25 μL. The PCR conditions were as follows: 94°С for 3 min, and 35 cycles (94°С for 40 s; 55°С for 30 s; and 72°С for 60 s) and 72°С for 7 min.

The amplicons were recovered using a 1% agarose gel and purified with the kit for DNA elution from agarose gel (Biosilica, Russia). The purified amplicons were ligated into a pAL-TA vector (Evrogen, Russia) with 1 U of T4 DNA Ligase (Thermo Fisher Scientific, UK). The construct was used for the transformation of the *E.coli* Top-10 competent cells, which were prepared according to the CCMB80 protocol [16]. The colonies with the target insert were selected using a blue-white selection with X-Gal/IPTG and PCR with primers to target the sequences. Plasmid DNA was extracted with the Biosilica kit for pDNA extraction (Biosilica, Russia). The sequencing was performed using an ABI PRISM Dye Terminator Cycle Sequencing ready reaction kit (Perkin Elmer Cetus, USA) with M13 primers and extra primers for the following target sequences: F-5UTRad-TTCTTCACACTAGGGCTGGT; R-5UTRad-CGCATAATAGCACAACCAGC; F-ex4-GTGGCAGTGGTAGTGGAAGT; and F-7ex-ACGCCGCTCAGATGAAGCAA.

### Diurnal quantitative expression of the photoperiod genes

Sonora, PSL2, “Ppd-m”, and “Ppd-w” plants were grown for 21 days after germination under controlled conditions in a climatic chamber Rubarth Apparate (RUMED GmbH) with short days (9 h of light, 20 °C). Three replicate samples from each genotype were harvested into liquid nitrogen at each three-hourly time point over 24 h since the beginning of the light period. RNA was extracted using the Plant RNA MiniPrep (Zymo Research, USA), followed by a DNase treatment with the RNase-Free DNase set (QIAGEN, Hilden, Germany). cDNA was synthesized using RevertAid First Strand cDNA Synthesis (Thermo Fisher Scientific, Lithuania) following the manufacturer’s protocol with 2 μg of total RNA as a template and Oligo(dT)_18_ as the primers. In total, 2 μl of the 20-fold dilution of the final cDNA were used for the following analysis.

For the expression analysis, previously published primers for the *TaFT-1, Ppd-1* [11], *Vrn-1* [17], *PhyA*, *PhyB* and *PhyC* [18] genes were used. The primers for the *FHY3/FAR1* were designed using the URGI: Traes_5BS_BCC406654.2 sequence. The primer sequences are as follows: F-TaFHY3/FAR1-5B- GCAAACGTCATCAGGATACA and R-TaFHY3/FAR1-5B- CCTCTTCTCAGCTTTACTTGC. The primers for the 18S rRNA gene [1] were used for normalization. The fluorescence data were collected using ABI 7500fast Real-Time PCR System (Applied Biosystems, Foster City, CA, USA) with SYBR Green I (Syntol, Russia) as the intercalating dye. The measurements were performed in three technical replicates. The reaction products were checked by a melting curve analysis and 2% agarose gel electrophoresis. The relative expression values were calculated according to the method proposed by Pfaffl [19]. The PCR efficiencies were determined using LinReg software [20]. The expression of target genes was normalized against 18S rRNA.

### Statistical analysis

ANOVA with a post hoc Tukey test was used to compare the expression levels. Correlations between the patterns of gene expression were calculated using Pearson coefficient (significance level *P* = 0.001).

### Bioinformatic analysis of the gene promoters

The PlantPAN 2.0 [13] database was used to determine the putative plant cis-acting regulatory elements. The 2000 bp upstream TSS (Transcription Start Site), first exon and first intron were analyzed for all sequences except for *TaFT-5D* due to the absence of the sequence of its upstream TSS in the databases.

## Results

### Sequence analysis of the photoperiod insensitive *Ppd-B1* allele

We have hypothesized that the *Ppd-B1* gene in the PI Sonora, “Ppd-m” and “Ppd-w” is characterized by not only an increased copy number but also by a nucleotide polymorphism in one of the copies because NILs “Ppd-m” and “Ppd-w” flowered slightly later than the line with the same background and introgressed *Ppd-D1a* gene [14]. Therefore, we analyzed the sequences of the *Ppd-B1* distinct copies. To investigate the possible polymorphisms, the amplicons that overlapped gene and its promoter were obtained and inserted into a vector to transform *E. coli* and distinct colonies that were regarded to have different copies, which were then sequenced and analyzed.

**Fig. 1:**
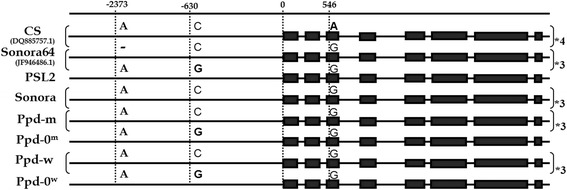
Scheme of the *Ppd-B1* gene features. Distances are presented in base pairs from the TSS (transcription start site). Black rectangles indicate exons. CS means Chinese Spring cultivar. Numbers on the right side of some sequences indicate number of gene copies in actual alleles

The results are presented in Fig. 1. Every line in our investigation differed by a single nucleotide indel in the promoter region (−2373 bp) of the *Ppd-B1* allele, which was detected in the cultivars Sonora64, Timstein (DQ885765.2), and Renan (DQ885764.2). Thus, the NILs and the parental cultivar Sonora had the same *Ppd-B1* intercopy region as cultivars Sonora64, Timstein and C591. However, the detected polymorphism provided a distinction among these *Ppd-B1* alleles.

The other SNP (−630 bp) allows for the distinction between the PI and PS NILs. Thus, “Ppd-m” and “Ppd-w” have “C” and the sister lines have “G” upstream the coding region. In exon 3 (+546 bp), the detected SNP distinguished the investigated sequence from another allele by the increased copy number (Chinese Spring).

### Diurnal expression analysis

To study the interactions between the *Ppd-B1a* PI allele and other genes that are important for wheat flowering, we used a qRT-PCR assay. We used 21-day-old plants from the late-flowering parental line PSL2, early flowering parental cultivar Sonora with the PI *Ppd-B1a* allele, and NILs “Ppd-m” and “Ppd-w”, representing PSL2 with different alleles at some loci, including *Ppd-B1a,* introgressed from Sonora.

**Fig. 2:**
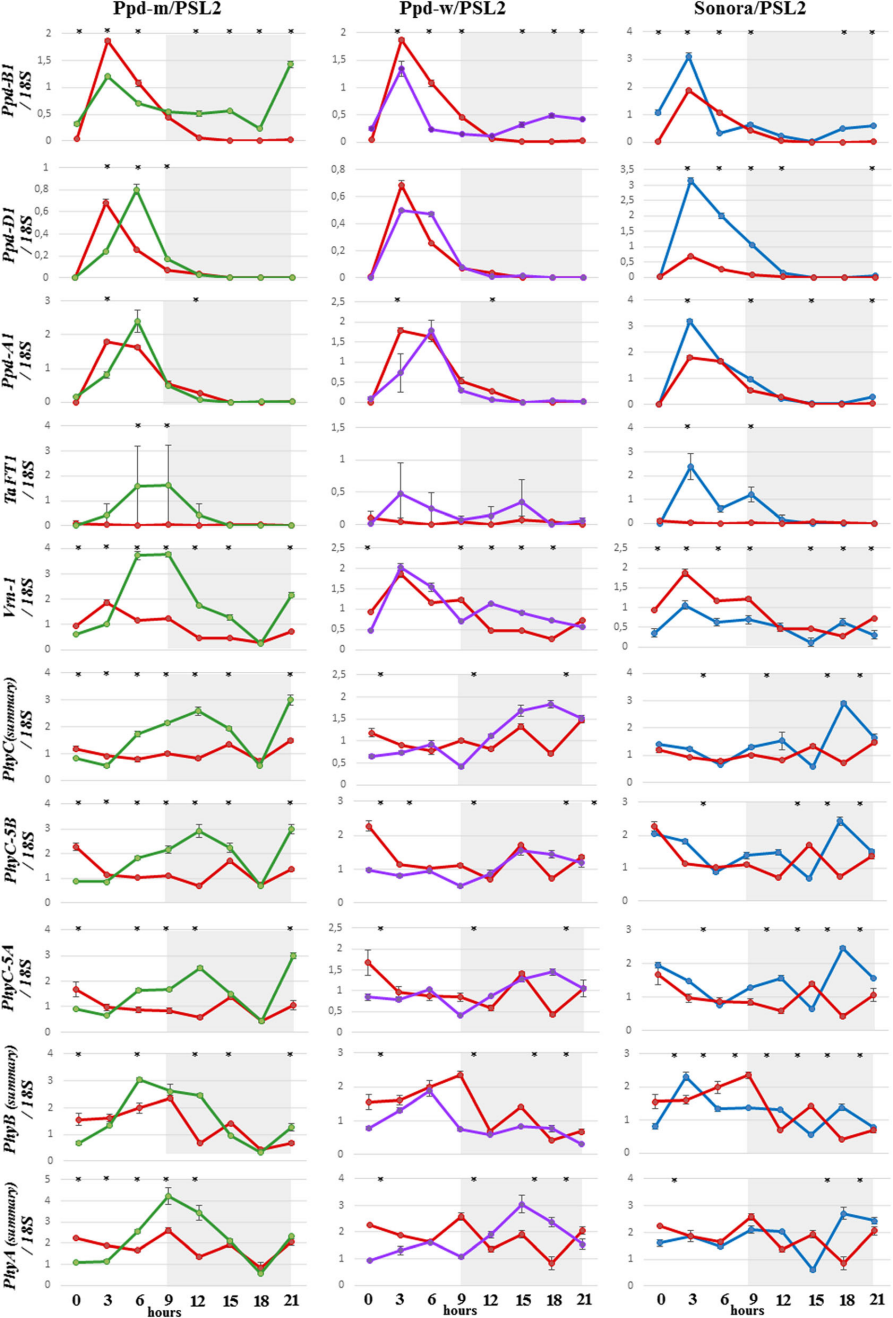
Patterns of the diurnal gene expression. Quantitative gene expression data from plants grown in short days (0–9 h light period) in а climatic chamber. Gray shadowing indicates the dark period (9–24 h). The graphs compare the expression between the photoperiod sensitive parental line PSL2 (red) and the photoperiod insensitive NILs (“Ppd-m” (green), “Ppd-w” (purple)) and parent Sonora (blue). Values are expressed as the relative levels normalized against 18S ribosomal RNA. Error bars indicate the SE of the means. Asterisks indicate significant (*P* < 0.05) differences in a one-way ANOVA with a post hoc Tukey test comparing the photoperiod insensitive NILs (“Ppd-m”, “Ppd-w”) and the parent Sonora with the photoperiod sensitive parental line PSL2 in each time point

The results of the diurnal expression analysis are presented in Fig. 2. *Ppd-B1* was expressed in the PI lines during the dark period but was not expressed in the photoperiod sensitive PSL2 as expected. The *TaFT1* gene was expressed only in the PI lines, supporting previous investigations [10, 11] in which the influence of the *Ppd-1* dominant alleles on *TaFT1* expression was shown.

The *Ppd-D1* expression was increased in Sonora compare to that in PSL2 and the NILs. This finding may be due to the 5 bp deletion in exon 7. The specificity of *Ppd-A1* is shown in the expression peak shift in “Ppd-m” and “Ppd-w” compared to that in Sonora and PSL2. The shift in the expression peaks in the “Ppd-m” line can be observed in *Ppd-D1*, *TaFT1*, *Vrn-1* and the other genes. The expression patterns of *Vrn-1* did not display any robust differences between Sonora and “Ppd-w” but was rather higher in Ppd-m. *PhyC* expression decreased since the beginning of the light period and displayed one or two peaks during the dark period. A tendency of an elevated expression during the dark period in the PI lines was observed. The expression patterns of *PhyB* and *PhyA* could be characterized by the two expression peaks as follows: one at the end of the light period and one during the dark period.

We analyzed the correlations (Additional file 1: Table S1) between the expression patterns of the genes involved in light perception (*PhyA*, *PhyB*, *PhyC*) and those involved in the flowering transaction (*Ppd-A1*, *Ppd-B1*, *Ppd-D1*, *Vrn-1*, *TaFT1*) separately during the dark and light periods in the photoperiod sensitive and insensitive lines.

The expression patterns of all the phytochromes significantly correlated with each other, indicating that common factors influence their expression. The *Ppd-1* genes also significantly correlated with each other (ρ = 0.55–0.85 in the photoperiod insensitive and ρ = 0.89–0.97 in the photoperiod sensitive lines). *TaFT1* expression correlated with the *Ppd-1* genes in the PI lines, and in PSL2, *TaFT1* was not expressed at all. We observed significant correlations between the expression levels of *TaFT1* and *PhyС-5A* (ρ = 0.75) and between *TaFT1* and *PhyC-5B* (ρ = 0.8) during the light period. The expression patterns of *Vrn-1* significantly correlated with the *Ppd-1* genes (ρ = 0.89–0.95) in PSL2. This finding may suggest that there are common factors influencing their expression. In the PI lines, *Vrn-1* correlated with all the phytochromes during the light (ρ = 0.65–0.79) and dark (ρ = 0.56–0.72) periods. These data are consistent with [21], in which the influence of *PhyC* and *PhyB* on *Vrn-1* expression was shown using RNA-Seq analysis. The correlation between *Ppd-B1* and *PhyC* (*PhyC* in combination and *PhyC-5B* and *PhyC-5A* individually) was significant during the dark period in the PI lines (ρ = 0.67).

### Features of the expression patterns in the lines with the photoperiod insensitive *Ppd-B1* allele

The expression peaks of all genes except for *Ppd-B1* were shifted in the “Ppd-m” line. The parental forms and “Ppd-w” did not display these expression patterns. Most likely, the genes located in the additional introgressions from Sonora, which are specific to “Ppd-m” but not “Ppd-w”, interact with the PSL2 background genes, which may result in such an effect. Both the “Ppd-m” and “Ppd-w” lines have introgression of *Ppd-B1a* from Sonora, but they differ by four days in their heading date under SD conditions (“Ppd-m” flowered earlier) [14]. “Ppd-m” has the following two supplemental loci from Sonora that were not found in “Ppd-w”: on chromosome 4D nearby the *Xbarc165*, on chromosome 5A nearby the *Xgwm154* and in the pericentromeric region of chromosome 5B (markers *Xbarc74*, *Xgwm67*, *Xgwm371*, and *Xgwm213*). QTLs associated with the heading date were previously identified in the 4D and 5B chromosomes. In chromosome 4D, the QTL was located in the region between markers *wPt8836* and *Xgwm165* [22]. No genes that are located in these areas are known to correspond to the heading time regulation.

**Fig. 3:**
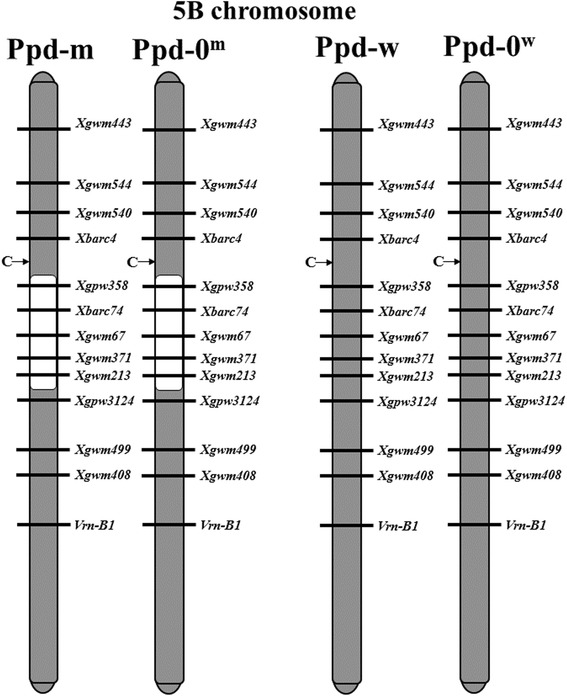
5B chromosome genotyping of NILs**.** Genotypes of the 5B chromosome of the near-isogenic “Ppd-m” and “Ppd-0^m^”, “Ppd-w” and “Ppd-0^w^” lines with introgressed chromosome regions of the Sonora variety (white color) into the genetic background of the recipient PSL2 parent (gray color). C indicates centromere region

In chromosome 5B, the heading date QTL was associated with the *Xgwm371* marker [23]. Later, a QTL associated with candidate genes *FHY3/FAR1*, *AP2/ERF* and *WRKY* was identified in the 5B pericentromeric region [24]. Therefore, chromosome 5B was genotyped using an additional set of SSR markers to ascertain the length of the introgression region. As a result, chromosome 5B in the “Ppd-w” and “Ppd-0^w^” lines was completely inherited from PSL2. In the line “Ppd-m”, there is an introgression from the early flowering parental Sonora precisely in the region that carries the *FHY3/FAR1* locus, while in “Ppd-w”, which flowers four days later than Ppd-m, does not have this introgression (Fig. 3). *FHY3/FAR1* is proposed as a good candidate to explain the difference in the heading date.

**Fig. 4:**
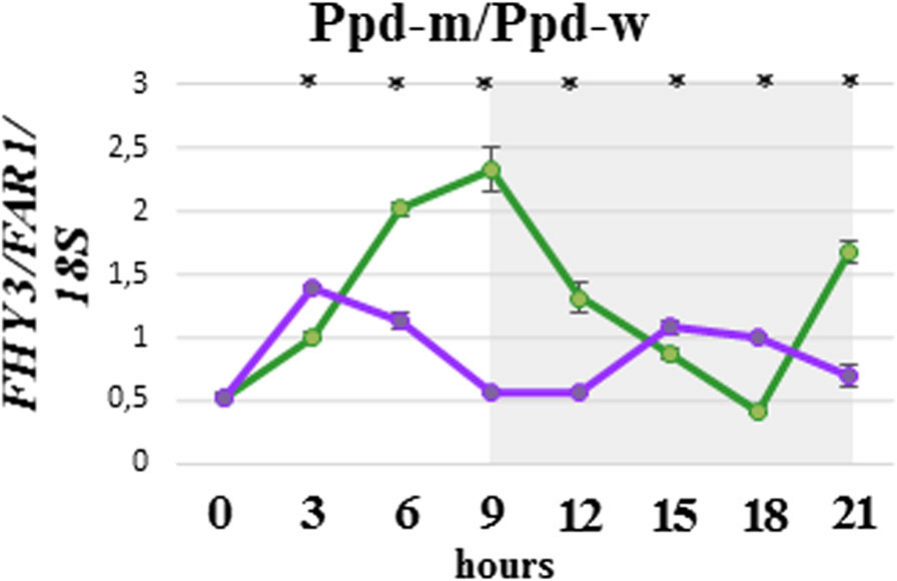
Patterns of the *FHY3/FAR1* diurnal expression. Quantitative gene expression data from plants grown in short days (0–9 h light period) in а climatic chamber. Gray shadowing indicates the dark period (9–24 h). The graphs compare the expression between the photoperiod insensitive NILs “Ppd-m” (green), “Ppd-w” (purple). Values are expressed as the relative levels normalized against 18S ribosomal RNA. Error bars indicate the SE of the means. Asterisks indicate significant (P < 0.05) differences in a one-way ANOVA with a post hoc Tukey test comparing the “Ppd-m” and “Ppd-w” in each time point

To examine whether there are any differences in *FHY3/FAR1* expression between lines “Ppd-m” and “Ppd-w”, we applied the diurnal expression analysis and demonstrated significant differences of *FHY3/FAR1* expression. Expression of this gene in “Ppd-m” was higher in 6–9 h after dawn indicating putative reason for the heading time variation (Fig. 4). *FHY3/FAR1* expression pattern correlated with phytochromes and *Vrn-1* at significant levels. In night *FHY3/FAR1* correlated with *Ppd-B1* in PI lines (“Ppd-m”, “Ppd-w” and Sonora).

### Sequence analysis of *Ppd-D1* and *Ppd-A1*

*Ppd-D1* of Sonora has a 5 bp deletion that is specific to haplotype IV based on the classification suggested by Guo [25]. Phenotypically, this haplotype is much weaker than haplotype I, which is represented by the dominant PI allele with a 2 kb deletion in the promoter region, but slightly reduces the heading time [25]. However, in contrast to [25], who showed that the expression of the *Ppd-D1* haplotype IV was only 5% higher, Sonora’s *Ppd-D1* expression was statistically significantly higher during the light period than the expression of the PSL’s allele (corresponding to haplotype II). During the dark period, no expression was observed. Most likely, the high expression of Sonora *Ppd-D1* can partially explain the fact that the PI lines flower 7–12 days later than Sonora, because their *Ppd-D1* alleles are inherited from PSL2.

The *Ppd-A1* sequence of PSL2 and the NILs has a SNP (A/C) that has not been previously described in any published *Ppd-A1* sequence. The comparison included 286 sequences of tetra- and hexaploid wheat varieties and was accomplished using the Blastn algorithm.

## Discussion

*Ppd-B1* is the only *Ppd-1* gene with a dominant PI allele that is associated with the copy number variation. However, little is known about the mechanisms underlying its misexpression. Previously, using near-isogenic lines resulting from a cross of the cultivars Sonora and PSL2, which contrast in flowering time, we have shown that the difference in flowering time was associated with an introgression on chromosome 2B between the markers *Xgwm148* and *Xgwm388* from the PI Sonora variety [14]. *Ppd-B1* is known to be located in this position [26]. Early flowering NILs do not have the PI *Ppd-B1a* allele with a 318 bp insertion in the promoter region that was identified by Nishida et al. [7]. Using Real-Time PCR, we revealed that the NILs and Sonora are characterized by increased copy numbers of *Ppd-B1* [14].

In this study, we have shown that *Ppd-B1a* in the PI lines is characterized by an increased number of copies and possesses an intercopy junction similarly to cultivars Sonora64, Timstein and C591. However, the investigated *Ppd-B1a* allele differs from Sonora64, Timstein and C591 allele by an indel in the promoter region.

We have also found two SNPs. The SNP in exon 3 (+546 bp) distinguished the investigated sequence from the Chinese Spring allele with an increased copy number.

The SNP (−630 bp) distinguished between the PI and PS NILs. This SNP (“G”) was previously detected in cultivars Recital, Paragon and Winter-Abukumawase [1, 8]. It is interesting to note that the *Ppd-B1* gene of the parental cultivar Sonora and all the other tetra- and hexaploid wheats is characterized by “C” in this position. Thus, “G” in this position is rare (4 cultivars, including PSL2). However, no association between photoperiod sensitivity and this SNP was shown [1, 7, 8].

To study the possible influence of the detected SNPs on the expression of *Ppd-B1a*, the interactions between the investigated *Ppd-B1a* allele and other wheat flowering genes and the activity of these genes in the presence of *Ppd-B1a*, we performed a diurnal expression analysis.

We analyzed the correlations between the expression patterns of genes involved in light perception (*PhyA*, *PhyB*, *PhyC*) and those involved in the flowering transaction (*Ppd-A1*, *Ppd-B1*, *Ppd-D1*, *Vrn-1*, *TaFT1*) separately during the dark and light periods in the photoperiod sensitive and insensitive lines.

*TaFT1* expression correlated with the *Ppd-1* genes in the PI lines, but in PSL2, *TaFT1* was not expressed at all. We identified the following cis-elements that corresponded to the RR (Response Regulator) or PRR (Pseudo Response Regulator) regulation: *ARR14*, *RR14*, *PRR4*, *APRR2*, *ARR1*, *RR1* (Myb/SANT, MYB, ARR-B families) and binding site ARR1AT. These findings propose the putative sites for the *TaFT1* regulation through the *Ppd-1* genes.

Significant correlations were found between the expression levels of *TaFT1* and *PhyС-5A* and *TaFT1* and *PhyC-5B* during the light period. During the night, the *TaFT1* expression level was very low, and phytochromes are inactive during the dark. It was suggested that phytochromes influence *TaFT1* expression. Altogether, this finding demonstrated that *Ppd-1* is important but not essential for flowering, and based on the triple *Ppd-1* loss-of-function mutant analysis [27], it was proposed that *TaFT1* is directly regulated by the phytochromes. Most likely, this process is performed with the assistance of *Ppd-1*. In the *TaFT1* promoter, the following binding sites for the transcription factor that is associated with the phytochrome regulation were found: *PIL5*, *POC1, PIF3*, *PAP3* (bHLH/bZIP), *FHY3/FAR1* (FAR1), *VOZ2* (VOZ), SORLIP1AT, SORLIP2AT, SORLREP5AT, SORLIP5AT, and RE1ASPHYA3. The expression correlation and the identification of TFBSs for Phytochrome-regulation in the *TaFT1* promoter suggest that phytochromes are other regulators of *TaFT1*.

We detected a significant correlation between *Ppd-B1* and *PhyC* (*PhyC* in combination and *PhyC-5B* and *PhyC-5A* individually) during the dark period in the PI lines.

Phytochromes express in the dark and produce inactive Pr (Red light-absorbing phytochrome) molecules [28, 29] that cannot influence the expression of other genes; thus, the correlation should imply that the expression of *Ppd-B1* during the night period positively influences *PhyC* expression, but *PhyC* does not influence *Ppd-1* during the night. Regarding *PhyA,* the same tendency can be observed, but no significant correlation was found. Because wild type *Ppd-1* reveals no expression during the dark period, it is unable to influence *PhyC* in general. We may hypothesize that *Ppd-1* influences *PhyC* even during the day period, but *PhyC* mRNA degradation in light conceals this putative effect. Previously, it was shown that the NILs with the PI *Ppd-1a* alleles (*Ppd-D1a* or *Ppd-B1a*) have an increased level of phytochrome protein compared to the sister lines with recessive *Ppd-1* alleles [30]. Thus, we suggest that the *Ppd-1a* genes may influence the *PhyC* expression directly or indirectly. To verify this hypothesis, we analyzed the promoter sequences of the *PhyC* genes to detect the possible binding sites for *Ppd-1*.

The binding sites for the response regulators (TFmatrixID_0348 for ARR2, RR2 and TF_motif_seq_0268 for ARR1AT) and the pseudo-response regulators (TF_motif_seq_0252 for APRR4) may be regulated by these pseudo-response regulators, such as *Ppd-1* genes. Some of these TFs correspond to phytochrome-regulation (*EPR1/RVE7*, *POC1/PIF3*, *FHY3/FAR1*, *SORLIP2AT*, and *SORLIP1AT*), suggesting their self-regulation. There were some other TFBSs that were associated with flowering or photoperiod regulation as follows: *GATA12, AtHB33, AtDOF1, SPL3, TEM1, STM, GBOX10NT, BS1EGCCR,* and *IBOXCORE.*

Taken together, the expression correlation data, the *PhyC* promoter analysis and the fact that there are phytochrome protein increments in the lines with the PI *Ppd-1a* alleles, as demonstrated by [30], suggest that *Ppd-B1a* is expressed during night period and positively regulates *PhyC* expression.

“Ppd-m” flowered four days earlier than “Ppd-w”. Using SSR genotyping, we demonstrated that the loci in the 5B pericentromeric region were different in the “Ppd-m” and “Ppd-w” lines. *FHY3/FAR1* is known to be located in this locus [24]. Binding sites for *FHY3/FAR1* were identified in the *Ppd-1*, *PhyC* and *TaFT1* promoters.

Using the diurnal expression analysis, we found that the *FHY3/FAR1* expression was higher in “Ppd-m” than in “Ppd-w”. *FHY3/FAR1* expression pattern correlated with phytochromes during the 24-h period in all the lines and with *Ppd-B1* in night in PI lines.

*FHY3/FAR1*, known to be involved in phytochrome signaling in *Arabidopsis* and rice [31, 32], control phytochrome accumulation through the *FHY1* regulation in *Arabidopsis* [33]. However, there is no data about *FHY3/FAR1* functions in wheat.

In wheat, the phytochromes (PhyB and PhyC) influence the *Ppd-1* expression [18, 21] and flowering time. *FHY3/FAR1*, which is involved in flowering time control, may contribute to this process. *Ppd-B1a* was suggested to increase *PhyC* expression. Thus, the data propose a bidirectional regulation between the *PhyC* and *Ppd-B1a* genes with a putative *FHY3/FAR1* contribution. This hypothesis requires further investigation and verification. The near-isogenic lines used in this study differ in the 5B pericentromeric region and are a good source for this work.

The lines with *Ppd-D1a* also demonstrated an increase in the phytochrome protein [30]. Therefore, this mechanism is proposed to be common for all *Ppd-1a* alleles. However, the regulation of *Ppd-B1* expression should be different.

Previously, it was suggested that a deletion in the promoter region of *Ppd-A1* and *Ppd-D1* causes the misexpression of the corresponding genes due to the disappearance of the CHE-binding site and impossibility of the repressor CHE (CCA1 HIKING EXPEDITION) to associate with its regulatory element [7]. Then, it could be hypothesized that an increase in the *Ppd-B1* copy number, which was shown to occur in its dominant alleles, results in a misexpression because of the increase in the *Ppd-1* genes with the same quantity of the repressor. However, in this case, an altered expression should be observed in all three *Ppd-1* genes. Previously, published data [11] demonstrated that the *Ppd-B1a* allele shows a misexpression by itself but does not influence *Ppd-A1* and *Ppd-D1* expression. Thus, about the hypothesis regarding the role of the *Ppd-B1* copy number against the repressor’s quantity should be rejected. It is interesting to note that there is no *Ppd-B1* allele with a deletion in the promoter region, while all PI alleles of *Ppd-A1* and *Ppd-D1* are characterized by deletions.

To identify the probable factors involved in *Ppd-B1* but not *Ppd-A1* and *Ppd-D1* regulation, we investigated the sequences of the promoter regions of these genes and determined (1) TFBSs (Transcription Factor Binding Sites) that are common to the promoters of all *Ppd-1* homoeologous genes and (2) TFBSs that are specific to *Ppd-B1*.

**Fig. 5:**
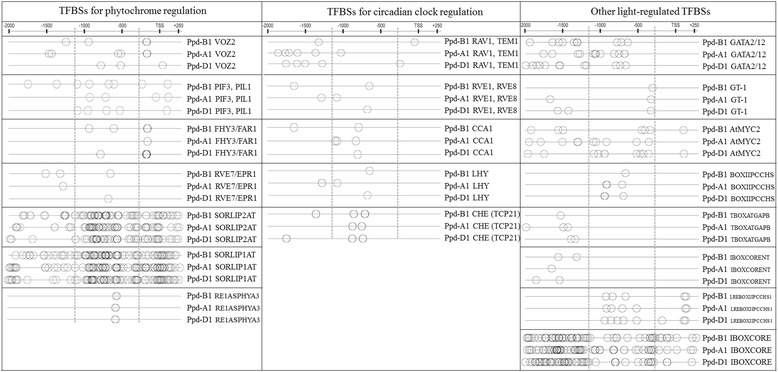
Groups of transcription factor binding sites that are common in the *Ppd-1* genes. Vertical dotted lines indicate the borders of a 900-bp region that is commonly deleted in the *Ppd-A1* and *Ppd-D1* photoperiod insensitive alleles [4, 7]. TSS indicates Transcription Start Site; the distances are presented in base pairs

To detect the TFBSs that are common to the *Ppd-1* genes in the A, B and D genomes, a PlantPan2 multiple promoter analysis was utilized. An in silico promoter sequence analysis revealed that that the *Ppd-1* promoters have many cis-acting elements that are associated with flowering that could be divided into the following three groups according to their input signal nature: phytochrome-regulated, circadian clock-regulated and other binding sites that are involved in light-regulated development. A detailed description of these transcription factors is summarized in Table 1. The positions of these cis-elements on the *Ppd-1* sequences are presented in Fig. 5.

**Table 1:** Characterization of the selected transcription factors and the TFBSs with unknown transcription factors that were identified in the promoter regions of the *Ppd-1* genes

Family	Transcription factors/TFBSs with unknown TF	Description
Phytochrome regulated transcription factors		
VOZ	VOZ2	*VOZ2* was identified as one of the highly conserved transcriptional factors in land plant genes that are PhyB-interacting factors [58].
bHLH	Phytochrome Interacting Factor3 (PIF3)	The G-box, CACGTG, is a target sequence for PIF3 [59]. PIF3 plays important roles in the Phy-mediated light responses. This factor can regulate the downstream genes either positively or negatively [60]. In *Arabidopsis,* PIF3 was suggested to play an important role in the control of flowering by the regulation of *CO* and *FT* gene expression [61].
bHLH	Phytochrome Interacting Factor3-Like5 (PIL1)	PIL5 interacts with the Pfr forms of Phytochrome A (PhyA) and Phytochrome B (PhyB) [62].
FAR1	Far-Red Elongated Hypokotyls3/Far-Red-Impaired Response1 (FHY3/FAR1)	Transcription factor FHY3/FAR1 modulates PhyA-signaling in higher plants [63]. Other investigations demonstrated that FHY3 plays a principal role in the circadian clock, heading date control and regulation of heading time through *ELF4* (*EARLY FLOWERING4*) [64].
Myb/SANT; MYB-related	RVE7/EPR1	*RVE7/EPR1* is regulated by both PhyA and PhyB and negatively regulates flowering [65].
–	SORLIP2AT and SORLIP1AT	Sequences over-represented in the light-induced promoters SORLIP2AT and SORLIP1AT were identified in the PhyA-induced promoters [66].
–	RE1ASPHYA3	RE1ASPHYA3 **(**RE1, putative repressor element) is a highly conserved motif in the most monocot *PhyA* promoters and is responsible for the Pfr-directed repression [67]; this motif was detected in certain other genes [68].
Circadian-clock regulated transcription factors		
AP2/RAV/B3	RAV1	*RAV1* is a negative component in the regulation of plant development [69].
AP2/RAV/B3	TEMPRANILLO1 (TEM1) TEMPRANILLO2 (TEM2)	*TEM1* and *TEM2* genes act as direct repressors of *FT* [70].
MYB-related	REVEILLE1 (RVE1)	RVE1 is a morning-phased transcription factor that integrates the circadian clock and auxin pathways [71].
Myb/SANT; MYB-related	REVEILLE 8 (RVE8)	*RVE8* promotes the expression of some evening element that contains clock genes and forms a negative feedback loop with *PRR5* [72, 73].
MYB	Circadian Clock Associated 1 (CCA1); Late Elongated Hypocotyl (LHY)	The MYB transcription factors *CCA1* and *LHY* are some of the key genes in the central oscillator of the plant circadian clock [74]. LHY and CCA1 negatively regulate *TOC1* expression.
TCP	CCA1 HIKING EXPEDITION (CHE)	The TCP transcription factor *CHE* is a clock component that is partially redundant with *LHY* in the repression of *CCA1* [75].
Other light regulated transcription factors		
GATA/tify	GATA2	GATA2 directly regulates genes that respond to light [76].
GATA/tify	GATA12	GATA12 is involved in the regulation of many light-responsive genes [77, 78].
MADF; Trihelix	GT-1	GT-1 may act as a light-responsive transcription factor [79].
bHLH	AtMYC2	AtMYC2 acts as a negative regulator of blue light–mediated photomorphogenic growth and blue and far-red-light–regulated gene expression [80].
–	BOXIIPCCHS	BOXIIPCCHS was suggested to be essential for light regulation [29, 81].
–	TBOXATGAPB	Mutations in TBOXATGAPB cause a reduction in light-activated transcription [82, 83].
–	IBOXCORENT	I-box core motif IBOXCORENT in the conserved DNA modular arrays is associated with the light-responsive promoter regions [84].
–	LREBOXIIPCCHS1	Light responsive element LREBOXIIPCCHS1, which was detected in parsley, is required for light responsiveness [85].
–	IBOXCORE	Conserved sequence in the 5′ UTR of light-regulated genes IBOXCORE may be involved in the regulation of transcription by light and the circadian clock. This sequence was detected in both monocots and dicots [86].

*PhyC* and *PhyB* were recently shown to regulate *Ppd-1* expression [18, 21]. These genes were suggested to be important regulators of the photoperiod sensitivity and flowering transaction in wheat [18, 21] and barley [34–36]. Although the chromosome position of *PhyA* in wheat was identified [37], no association between these loci and flowering was described. However, *PhyA* is an important regulator of flowering time in certain other species, such as *Arabidopsis* [38, 39]. Therefore, an investigation of the *PhyA* sequence and functions in cereals may complement the mechanisms of the photoperiod sensitivity and flowering. Here, we may hypothesize that *Ppd-1* expression is regulated by the phytochromes with the assistance of transcription regulators, such as *PIF3*, *PIL5*, *FHY3/FAR1*, *RVE7/EPR1*, and *VOZ2,* and certain TFBSs with unknown transcription factors, such as SORLIP2AT, SORLIP1AT, and RE1ASPHYA3.

The *Ppd-1* genes were suggested to be regulated by the circadian oscillator in wheat [18] and barley [40]. In barley, some genes that are involved in this process are known, while in wheat, no putative circadian affecters are proposed. We identified the TFBSs that might be involved in the circadian clock regulation of the *Ppd-1* genes as follows: *RAV1*, *TEM1*, *TEM2*, *RVE1*, *CCA1*, *LHY*, and *CHE*.

The *Ppd-1* activation by *PhyC* was proposed to be light sensitive throughout the day [41]. Most likely, this process can be mediated by some of the following detected elements: *GATA2*, *GATA12*, *GT-1*, and *AtMYC2* and some binding sites with unknown transcription factors, such as BOXIIPCCHS, TBOXATGAPB, IBOXCORENT, LREBOXIIPCCHS1, and IBOXCORE.

**Fig. 6:**
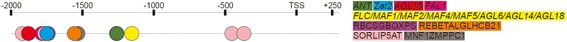
Groups of transcription factor binding sites that are specific to *Ppd-B1*. TSS indicates Transcription Start Site; the distances are presented in base pairs. Different colors designate different TFBSs or TFBSs with unknown TF

Binding sites that are specific to *Ppd-B1* and not *Ppd-A1* and *Ppd-D1* are likely associated with the circadian rhythms and flowering time and regulatory elements from the following families: AP2 (*ANT*), C2H2 (*Zat12*), MADS box/MIKC (*AGL19*, *FLC*, *MAF2*, *AGL69*, *AGL68*, *FLM*, *AGL6*, *AGL18*, *AGL14*, and *AGL15*), and Lyase Aromatic (*PAL1*) and TFBSs with unknown TF RBCSGBOXPS, REBETALGLHCB21, LEAFYATAG, SORLIP5AT, and MNF1ZMPPC1. A detailed characterization of these transcription factors is presented in Table 2. The positions of these TFBSs on the *Ppd-B1* sequence are presented in Fig. 6.

**Table 2:** Characterization of the selected transcription factors and the TFBSs with unknown transcription factors that were identified in the promoter regions of *Ppd-B1*

Family	Transcription factors/TFBSs with unknown TF	Description
AP2	AINTEGUMENTA (ANT)	ANT initiates floral organ development [87]. It was shown to play a critical role in regulating the ovule and female gametophyte development [88].
C2H2	RESPONSIVE TO HIGH LIGHT 41 (Zat2)	*Zat12* was originally isolated as a light stress-response cDNA [89]; then, it was suggested to be able to regulate transcripts involved in the response to high-light, cold and oxidative stress [90].
MADS box/MIKC	AGL19	AGL19 controls (promotes) flowering downstream of a cold-perception pathway and acts independently of FT and SOC1 [42].
MADS box/MIKC	FLOWERING LOCUS C (FLC)	FLC acts as an inhibitor of flowering [45].
MADS box/MIKC	MAF2 (AGL31)	*MAF2* (*AGL31*), a paralog of *FLC,* is another flowering repressor that acts in non-inductive photoperiods [46, 47].
MADS box/MIKC	AGL69 (MAF5)	*MAF5* is normally repressed. Overexpression of *MAF5* under a non-inductive day length causes late-flowering [48].
MADS box/MIKC	AGL68 (MAF4)	MAF4 represses the transition to flowering [49, 50].
MADS box/MIKC	FLM (AGL27, MAF1)	FLM acts as a flowering inhibitor [51].
MADS box/MIKC	AGL6	AGL6 was suggested to be able to act as a flowering repressor or activator, depending on the context [43].
MADS box/MIKC	AGL14 (XAANTAL2, XAL2)	XAL2 is essential for flowering induction. XAL2 promotes flowering in response to different signals and is important for the maintenance and differentiating of flowering meristems [44].
MADS box/MIKC	AGAMOUS-like 15 (AGL15)	AGL15 and AGL18 are floral transition repressors. The *agl15 agl18* mutants were characterized by a partial suppression of the photoperiod pathway [52].
MADS box/MIKC	AGAMOUS-like 18 (AGL18)	
Lyase Aromatic	Phenylalanine Ammonia-Lyase (PAL1)	PAL1 is a light response element. These motifs are conserved at similar positions in several elicitor or light-responsive genes from different species [91].
–	RBCSGBOXPS	RBCSGBOXPS binding site, identified in *Parsley*, is involved in light responsiveness [92].
–	REBETALGLHCB21	REBETALGLHCB21, first found in the *Lemna dibba Lhcb* genes, is necessary for phytochrome regulation. These elements are likely to function by repressing the promoter activity in the dark [93].
–	SORLIP5AT	SORLIP5AT are PhyA-induced motifs that are overrepresented in light-induced genes. These elements, which predominate in the early responsive promoters, are more likely to have the fewest steps in the signal transduction cascade to gene expression [66].
–	MNF1ZMPPC1	MNF1ZMPPC1 is involved in the light-dependent transcriptional control of gene expression [94].

The transcription factors that were associated with the flowering transaction included *MADS box* and the *MIKC* family genes. Some of these transcription factors contribute to flowering induction, and others contribute to flowering repression. For example, *AGL19*, *AGL6* and *AGL14* are positive regulators of the flowering transition [42–44]. In contrast, the majority of the identified MADS transcription factors, i.e., *FLC*, *MAF2*, *AGL69*, *AGL68*, *FLM*, *AGL15* and *AGL18*, negatively regulate the transition from vegetative to reproductive development [45–52]. Gu et al. [50] demonstrated that *FLC*, *MAF3 (AGL70)*, *FLM (MAF1,AGL27)*, *MAF2 (AGL31)* and *MAF4 (AGL69)* interact with each other and form nuclear complexes that are responsible for flowering repression.

Most of these TFs contribute to the same binding site, TFmatrixID_0503, except for *AGL15,* which is associated with the TF_motif_seq_0105 sequence. Motif TFmatrixID_0503 corresponded to the *MADS* transcription factors not only in *Arabidopsis* but also in *Brachpodium distachyon*, *Oryza sativa* and *Sorgum bicolor*. These transcription factors were shown to be involved in the flowering transaction and flowering time formation in rice [53, 54]. For example, *OsMADS50* is a positive regulator of flowering, and *OsMADS56* negatively influences the flower transition [54]. *OsMADS7* and *OsMADS8* are involved in flowering time modulation [55]. *MADS* genes were identified in wheat [56]. Some of them are homologs of rice *OsMADS8* (*OsMADS24*) and *OsMADS7* (*OsMADS45*), which can influence the flowering time [57]. However, no data are available regarding the binding sites for the wheat *MADS* genes; thus, we can only propose that their association is at the same sites as those for the *Arabidopsis* and rice *MADS* genes.

Based on the data regarding the regulatory elements in the *Ppd-B1* promoter and the common TFs in the homoeologous *Ppd-1* genes, we may propose that the *MADS* genes play a major role in the misexpression of *Ppd-B1a* with an increased number of copies. Many of these genes are known to be flowering repressors. Most likely, *Ppd-B1* with an increased copy number continues to express during the night period; thus, the quantity of the repressors remains the same.

Thus, we have identified a set of putative transcription factors that regulate all homoeologous *Ppd-1* genes. We divided these transcription factors into three groups according to the input signal as follows: phytochrome-regulated, circadian clock-regulated and other light-regulated. However, there are several *Ppd-B1* specific factors and *MADS* genes that are known to be flowering repressors and are most likely *Ppd-B1* regulators. Our future prospects include the verification of the involvement of the detected transcription factors in *Ppd-B1* regulation, and the discussed NILs are a relevant model for such studies.

## Conclusions

The results of this study suggest that there is a positive bidirectional regulation of *Ppd-B1a* and *PhyC* with an *FHY3/FAR1* contribution. The bidirectional regulation can be proposed for *Ppd-A1a* and *Ppd-D1a*. Using in silico analysis, we demonstrated that the specificity of the *Ppd-B1* regulation compared to that of homoeologous genes involves not only a copy number variation but also distinct regulatory elements.

## Additional files

Additional file 1: Table S1. Correlation coefficients of the expression patterns of the flowering genes. Bold font indicates significant values (*P* = 0.001). PI means Photoperiod Insensitive and PSL means Photoperiod Sensitive samples. (XLSX 26 kb)

## Abbreviations

AGL: Agamous-Like
ANT: Aintegumenta
*ARR*: *Arabidopsis Response Regulator*
CCA1: Circadian Clock Associated 1
cDNA: DNA complementary to RNA
CHE: CCA1 Hiking Expedition
CNV: Copy number variation
*FHY3/FAR1*: *Far-Red Elongated Hypokotyls3/Far-Red-Impaired Response1*
FLC: Flowering Locus C
FLM: Flowering Locus M
LHY: Late Elongated Hypocotyl
MAF: Mads Affecting Flowering
MITE: Miniature inverted-repeat transposable element
NILs: Near-Isogenic Lines
PAL1: Phenylalanine Ammonia-Lyase
*Phy*: *PhytochromeA*
PI: Photoperiod insensitive
PIF3: Phytochrome nteracting Factor3
PIL1: Phytochrome Interacting Factor3-Like5
*Ppd-1*: *Photoperiod-1*
*PRR*: *Pseudo-Response Regulator*
PS: Photoperiod sensitive
PSL2: Photoperiod Sensitive Line 2
qPCR: Quantitative PCR
QTLs: Quantitative Trait Loci
RAV1: Related to ABI3/VP1
RE1ASPHYA3: RE1 putative repressor element
RR: Response Regulator
rRNA: DNA coding for rRNA
RVE1: Reveille1
RVE7/EPR1: Reveille 7/Early Phytochrome Responsive 1
RVE8: Reveille8
SD: Short day
SORLIP2AT: SORLIP1AT: SORLIP5AT: Sequences over-represented in the light-induced promoters
SSR: Simple Sequence Repeat
*TaFT1*: *Triticum aestivum Flowering Locus T*
TEM1: Tempranillo1
TEM2: Tempranillo2
TFBS: Transcription Factor Binding Sites
TSS: Transcription Start Site
VOZ2: Vascular Plant One–Zinc Finger1
*Vrn-1*: *Vernalization-1*
Zat2: Responsive To High Light 41
